# The road to success: drawing parallels between 'road' and 'research data' infrastructures to foster understanding between service providers, funders and policymakers

**DOI:** 10.12688/f1000research.128167.1

**Published:** 2023-01-23

**Authors:** Rob W.W. Hooft, Corinne S. Martin

**Affiliations:** 1 Dutch Techcentre for Life Sciences, Utrecht, 3521 AL, The Netherlands; 2 ELIXIR Hub, Hinxton, Cambridge, CB10 1SD, UK

**Keywords:** Research Data, Research Infrastructure, Infrastructure Funding, Sustainability

## Abstract

**Background:** The work of data research infrastructure operators is poorly understood, yet the services they provide are used by millions of scientists across the planet.

**Policy and implications:** As the data services and the underlying infrastructure are typically funded through the public purse, it is essential that policymakers, research funders, experts reviewing funding proposals, and possibly even end-users are equipped with a good understanding of the daily tasks of service providers.

**Recommendations: **We suggest drawing parallels between research data infrastructure and road infrastructure. To trigger the imagination and foster understanding, this policy brief contains a table of corresponding aspects of the two classes of infrastructure.

**Conclusions:** Just as economists and specialist evaluators are typically brought in to inform policies and funding decisions for road infrastructure, we encourage this to also be done for research infrastructures

## Introduction

Data-intensive research depends on data and data services, such as databases, software and tools, and standards. These are often made available to end-users through research infrastructure. Such a research infrastructure for biological data and bioinformatics service in Europe is ELIXIR. ^1^

As is common for many types of infrastructure (especially those that are free at the point of use), the existence of research infrastructure, like the services provided by ELIXIR, is often taken for granted by their users. Their importance is only noticed when they are (temporarily) unavailable, or worse, could disappear due to discontinued funding. ^2^^,^^3^ In the Kano model, infrastructure services are must-be qualities: their proper functioning does not make the users happy, but service disruptions are strong dissatisfiers.

As research infrastructures and their services are typically funded through the public purse, it is essential that policy makers, research funders, experts reviewing funding proposals, and possibly even end-users are equipped with a good understanding of the daily tasks of service providers. We have noticed that this is not often the case, and this becomes an issue when this lack of understanding affects the funding of research infrastructures. To foster better understanding, this policy brief provides a comparison table of features of data infrastructure and their relatable counterparts in road infrastructure. We believe that using this approach can help, first because the change in mind frame makes it possible to see consequences of certain choices more clearly, especially those linked to funding of research infrastructures. Second, many people, even those working in research, are much more accustomed to road infrastructure setup and road infrastructure disruptions than to research infrastructure setup and research infrastructure disruptions; this increased familiarity increases the chances that consequences of policy decisions are foreseen.

For the actual feature comparison, see Table 1.

**Table 1.** Comparable concepts in research data infrastructure and road infrastructure.

Data infrastructure	Road infrastructure
Data service	Road
User of a data service/Researcher	Car driver
Research project	Car
Cutting edge research project	Expensive car
Project	Trip, journey
The one who develops and operates a service, service provider	Road construction and maintenance company
Flexible data service	Road suitable for cargo and people
Hack something together yourself	Driving your own Jeep from Manchester to Cambridge, possibly off-road
Using a well-documented and well-maintained existing service	Driving the motorway from Manchester to Cambridge in a mid-class comfortable car
Services that the user pays for	Toll road
Centrally financed service that therefore is oversubscribed/abused (e.g. free repository where someone dumps 1TB of data)	Traffic Jam
Services that can be used by anyone, the user does not need to be part of the infrastructure that provides them	Public roads
Interoperable services	Road intersections, motorway interchanges
Non-interoperable services	Crossing roads without intersections
Efficiency of a well-thought-out service	Speed of a Ferrari on an asphalt road
Efficiency of letting everyone figure things out themselves	Speed of a Jeep on rough terrain
Thinking data infrastructure is too expensive (or lacking sufficient accommodation of one's specific needs) and preferring to build everything yourself to exact specifications; underestimating the value of services (e.g. 24/7 support) and underestimating the real cost of building everything yourself (excluding e.g. energy bills, and the fact that the postdoc employed for the task is not productive for work while making weekly backups)	Not being willing to pay road taxes or to pay another centralised tax system (or complaining that you have to walk the last bit from the parking lot), underestimating the value provided (to its users) by the road infrastructure and underestimating the cost incurred to get exactly from A->B without roads (i.e. using an all-terrain car), e.g. forgetting that there won't be any gas stations along the way either
What can be realistically achieved in a scientific project	How far you can travel in a day
ELIXIR's visibility (i.e. prominence) in biology	Public knowledge about the companies building and maintaining roads
ELIXIR's visibility in bioinformatics	Knowledge in the government about the companies building and maintaining roads
Not knowing whether the infrastructure will live long enough to serve your entire project (and its successor), and therefore being coerced into building it yourself	Not knowing whether the roads will be maintained for 10 years, and therefore better off buying a Jeep than either a Ferrari or a Volkswagen
Infrastructure investing in services based on community needs	Government prioritising road investments based on transport needs
Asking a research infrastructure provider what scientific breakthroughs the infrastructure will be making. An RI facilitates/enables breakthroughs (as well as enabling more routine research to be carried out) but does not make them itself nor predict what they will be	Asking a road construction/maintenance company how much faster the distance will be travelled. The road enables road users to travel at average and perhaps faster speeds, but the road construction/maintenance company cannot predict what cars they will use and what they will carry
Making the requirement that a funding proposal for "infrastructure" includes scientific breakthroughs to happen within a set timeline, instead of enabling routine and breakthrough research as long as the infrastructure is useful	Asking a road company to deploy a combination of a very fast asphalt depositing machine, a Ferrari, and a system to clean up the road immediately afterwards
Asking top scientists to review proposals for research infrastructure funding. The services that would be offered by the infrastructure would be "competing" with what they (and only similar top scientists) can get done in their own labs	To ask those who have expensive Jeeps whether they approve the construction of a public road. They and others with off-road cars do not see the need for roads, they can get along just fine without them

## Policy outcomes and implications

The comparison between research data infrastructure and road infrastructure has many hooks to support productive discussions, and decisions, on research infrastructure funding and sustainability governance. Given that we, the co-authors, all work in research infrastructures, there is an inherent bias in the approach presented, just like when Sutherland *et al.* ^4^ published their “20 things politicians need to know about science”. Yet, we also do not intend to be counterproductive: policymaking is complex and multifaceted, as astutely explained in “ 20 things scientists need to know about policy”. The comparison table is simply our contribution to fostering longer-term sustainability of infrastructures that already exist, that are widely used across the world, and that have typically received significant public financing over the years.

Furthermore, the comparison table is likely to support efforts of both research infrastructure operators and policymakers in more accurately conveying to taxpayers the public value of research infrastructures, in addition to their role as enablers of scientific discovery and applications of societal benefit. The word ‘enablers’ is perhaps the most important message to convey: just like a road enables travel (and a research infrastructure enables research), it is questionable whether it is right to ask the road construction/maintenance company (and the research infrastructure operator) whether the road (and the research infrastructure) bring(s) value to society. Economists and evaluation specialists are very well placed and qualified (and likely unbiased) to answer complex questions around the public value of financing roads and research infrastructures. ^5^^,^^6^

## Actionable recommendations

***Recommendation 1***: When formulating opinions and decisions on research data infrastructure funding and sustainability governance, compare them with that of road infrastructure. The change of frame may bring new insights.

***Recommendation 2***: Consider informing policies and funding decisions relating to existing and future research infrastructure with support from economists and specialist evaluators.

## Conclusion

We welcome any additional rows for the comparison as well as discussion on improving the existing table in the original Google Document. We have found it difficult to broaden the set of comparisons to also include a sustainable travel angle (e.g. examples covering public transport versus private car travel). Considering the climate emergency, this would be a useful and still relatable expansion of the approach.

## Data Availability

No data are associated with this article
