# The road to success: drawing parallels between 'road' and 'research data' infrastructures to foster understanding between service providers, funders and policymakers

**DOI:** 10.12688/f1000research.128167.2

**Published:** 2023-08-22

**Authors:** Rob W.W. Hooft, Elaine Harrison, Corinne S. Martin

**Affiliations:** 1 Dutch Techcentre for Life Sciences, Utrecht, 3521 AL, The Netherlands; 2 ELIXIR Hub, Hinxton, Cambridge, CB10 1SD, UK

**Keywords:** Research Data, Research Infrastructure, Infrastructure Funding, Sustainability

## Abstract

**Background:** The work of data research infrastructure operators is poorly understood, yet the services they provide are used by millions of scientists across the planet.

**Policy and implications:** As the data services and the underlying infrastructure are typically funded through the public purse, it is essential that policymakers, research funders, experts reviewing funding proposals, and possibly even end-users are equipped with a good understanding of the daily tasks of service providers.

**Recommendations: **We suggest drawing parallels between research data infrastructure and road infrastructure. To trigger the imagination and foster understanding, this policy brief contains a table of corresponding aspects of the two classes of infrastructure, and a table of policy implications.

**Conclusions:** Just as economists and specialist evaluators are typically brought in to inform policies and funding decisions for road infrastructure, we encourage this to also be done for research infrastructures

## Introduction

Data-intensive research depends on data and data services, such as databases, software and tools, and standards. These are often made available to end-users through research infrastructure. Such a research infrastructure for biological data and bioinformatics service in Europe is ELIXIR. ^1^

As is common for many types of infrastructure (especially those that are free at the point of use), the existence of research infrastructure, like the services provided by ELIXIR, is often taken for granted by their users. Their importance is only noticed when they are (temporarily) unavailable, or worse, when they disappear due to discontinued funding. ^2^ ^,^ ^3^ In the Kano model, infrastructure services are must-be qualities: their proper functioning does not make the users happy, but service disruptions are strong dissatisfiers.

As research infrastructures and their services are typically funded through the public purse, it is essential that policy makers, research funders, experts reviewing funding proposals, and possibly even end-users are equipped with a good understanding of the daily tasks of service providers. We have noticed that this is not often the case, and this becomes an issue when this lack of understanding affects the funding of research infrastructures (funding decisions are typically made based on scientific advice to funding bodies). To foster better understanding, this policy brief provides a comparison table of features of data infrastructure and their relatable counterparts in road infrastructure ( Table 1).

**Table 1.** Key features of data infrastructure and their relatable counterparts in road infrastructure.

Data infrastructure	Road infrastructure
Data service	Transport infrastructure, such as a road
Researcher using a data service for a project	Car driver using a road for a journey
The service provider who develops and operates a data service	Road construction and maintenance company
Flexible/generic data service	Road suitable for most user types, such as cargo and people
Hacking a data service solution	Driving your own Jeep from London to Cambridge, off-road if needs be
Using a well-documented and well-maintained data service	Driving a well-serviced motorway from London to Cambridge in a mid-class comfortable car
Data service that users need to pay for	Toll road
“Free at the point of use” data service (e.g. centrally financed) with the potential to be oversubscribed or dominated by a subset of users by dumping 1TB of data	Toll-free road with the potential of becoming overcrowded/jammed and dominated by car drivers (in preference to mass transit such as buses, emergency vehicles, non-motorised users such as cyclists)
Data services that can be used by anyone (i.e. users do not need to be a member of the infrastructure	Public roads
Interoperable services	Being able to complete a journey in one vehicle by using roads maintained by different companies and through different countries
Non-interoperable services	A journey being interrupted by the vehicle not being suitable for a new road surface or not being allowed over a country border
Using a well designed data service	Driving your car on an well-marked asphalt road
Creating your own solution without access to common reference standards and guidelines	Building a rough and ready vehicle to carry you across uneven terrain
What can be realistically achieved in a scientific project	How far you can travel in a day
Life scientists’ awareness of ELIXIR’s role in creating and maintainting an infrastructure to support life science research	Public knowledge about the companies building and maintaining roads
Awareness in funding bodies of ELIXIR’s role in creating and maintainting an infrastructure to support life science research	Knowledge in national governments about the companies building and maintaining roads

We believe that this approach can help, firstly because the change in mindset makes it possible to see consequences of certain choices more clearly, especially those linked to funding of research infrastructures. Secondly, many people (decision makers and those influencing the decisions), even those working in research, are much more accustomed to road infrastructure setup and road infrastructure disruptions than to research infrastructure setup and disruptions; this increased familiarity increases the chances that consequences of policy decisions are foreseen.

## Policy outcomes and implications

The comparison between research data infrastructure and road infrastructure has many hooks to support productive discussions, and decisions, on research infrastructure funding and sustainability governance. Some examples of policy implications following from the comparison are given in Table 2.

**Table 2.** Policy implications of the comparison between data infrastructure road infrastructure.

Data infrastructure	Road infrastructure
Thinking data infrastructure is too expensive (or lacking sufficient accommodation of one's specific needs) and preferring to build everything yourself to exact specifications; underestimating the value of services (e.g. 24/7 support) and underestimating the real cost of building everything yourself (excluding e.g. energy bills, and the fact that the postdoc employed for the task is not productive for research work while making weekly backups and troubleshooting issues)	Not believing in road taxes nor other centralised tax systems (or complaining that you have to walk the last bit from the parking lot), underestimating the value provided (to its users) by the road infrastructure and underestimating the cost incurred to get exactly from A->B without roads (i.e. using an all-terrain car), e.g. forgetting that there won't be any gas stations or other support services along the way either
Not knowing whether the data infrastructure will be sustained for the life of your project (and its successor), and therefore building a makeshift infrastructure yourself	Not knowing whether the roads will be maintained for 10 years, and therefore planning your vehicles around off-road travel
Infrastructure investing in services based on community needs	Government prioritising road investments based on transport needs
Asking a research infrastructure provider what scientific breakthroughs the infrastructure will be making. A RI facilitates/enables breakthroughs (as well as enabling more routine research to be carried out) but does not make them nor predict what they will be	Asking a road construction/maintenance company where it will be driving its own cars on the new road, rather than asking what new economic activity the new road will facilitate
Requiring that a funding proposal for "infrastructure" the enables scientific breakthroughs during the lifetime of the grant	Giving a road construction company money to build a road only if they guarantee it will meet a threshold of user journeys during the 3 years it takes to build
Asking top scientists to review proposals for research infrastructure funding. The services that would be offered by the infrastructure would be competing with what they (and only similar top scientists) could achieve in their own labs	Asking those who own expensive Jeeps whether they approve the construction of a public road. They and others with off-road cars do not see the need for roads, they can get along just fine without them

Given that we, the co-authors, all work in research infrastructures, there is an inherent bias in the approach presented, just like when Sutherland *et al.* ^4^ published their “20 things politicians need to know about science”. However, we hope our thoughts have practical use: policymaking is complex and multifaceted, as astutely explained in “ 20 things scientists need to know about policy”. The comparison tables are simply our contribution to fostering longer-term sustainability of infrastructures that already exist, that are widely used across the world, and that have typically received significant public financing over the years.

Furthermore, the comparison tables are likely to support efforts of both research infrastructure operators and policymakers in more accurately conveying to taxpayers the public value of research infrastructures, in addition to their role as enablers of scientific discovery and applications of societal benefit. The word ‘enablers’ is perhaps the most important message to convey: just like a road enables travel (and a research infrastructure enables research), it is questionable whether it is right to ask the road construction/maintenance company (and the research infrastructure operator) whether the road (and the research infrastructure) brings value to society. Economists and evaluation specialists are very well placed and qualified (and likely unbiased) to answer complex questions around the public value of financing roads and research infrastructures. ^5^ ^,^ ^6^

## Actionable recommendations

***Recommendation 1***: When formulating opinions and decisions on research data infrastructure funding and sustainability governance, compare them with that of road infrastructure. The change of frame may bring new insights.

***Recommendation 2***: Consider informing policies and funding decisions relating to existing and future research infrastructure with support from economists and specialist evaluators.

## Conclusion

We welcome any additional ideas for the comparison as well as discussion on improving the existing tables as comments to this paper. For instance, the parallels could be improved by considering other infrastructures delivering public services, such as water supply and sewage systems. We tried, but found it difficult, to broaden the set of comparisons to also include a sustainable travel angle (e.g. examples covering public transport versus private car travel). Considering the climate emergency, this would be a useful and still relatable expansion of the approach.

## Data availability

No data are associated with this article.
