# Supplementary material for: The road to success: drawing parallels between 'road' and 'research data' infrastructures to foster understanding between service providers, funders and policymakers
Source: F1000Res. 2023 Aug 22;12:ELIXIR-88. Originally published 2023 Jan 23. [Version 2] doi: 10.12688/f1000research.128167.2 (PMC10102711; doi:10.12688/f1000research.128167.2)
Supplement: Peer review history

# Peer review: Reviewer response for version 2

**DOI:** 10.5256/f1000research.153851.r198831

**Published:** 2023-08-23

**Authors:** Frédéric Sgard

**Affiliations:** 1 OECD, Paris, France

No new comment.

Does the paper provide a comprehensive overview of the policy and the context of its implementation in a way which is accessible to a general reader?

Yes

Is the discussion on the implications clearly and accurately presented and does it cite the current literature?

Yes

Are the recommendations made clear, balanced, and justified on the basis of the presented arguments?

Yes

Reviewer Expertise:

Science policy

I confirm that I have read this submission and believe that I have an appropriate level of expertise to confirm that it is of an acceptable scientific standard.

# Peer review: Reviewer response for version 1

**DOI:** 10.5256/f1000research.140729.r161289

**Published:** 2023-04-13

**Authors:** Silvia Vignetti

**Affiliations:** 1 CSIL, Milan, Italy

This article sheds light on the important and often poorly understood world of research infrastructures providing data and other research services to scientific communities around the world to perform excellent science. It does so by showing the features a research infrastructure has with a road infrastructure, with the aim of making clear why and how public funding must be understood and justified.

 The article is well-written and to some extent illuminating. The parallel with road infrastructures is fitting quite well with the purpose of the authors. The parallel may go even further in depicting the parallels with other infrastructures delivering public services such as water supply, sewage system and so on. The article however does not fully exploit the potential of this parallel and remains a bit open and generic in the explanations.

 First, the table, which is pretty much the core of the article, presents some elements that should be better qualified, explained and perhaps even reconsidered in a few cases (e.g. " *data service*" corresponds perhaps more to 'transport service' than to " *road*"; it is not clear why " *research project*" corresponds to " *car*" and " *project*" to " *trip, journey*"; " *oversubscribed service*" may be 'crowded road' more than " *traffic jam*"), so it would be better to add a short explanation or a narrative section where the content of the table is described, justified and put in context.

 Second, the policy options and implications do not come immediately from the description of the main common features, especially for those who are not familiar with both research and more traditional infrastructures. Perhaps the table could be split in two to distinguish the key features (first half) from the policy implications on their funding and public justification (second half more or less). 

 Overall, the article is very interesting and well-conceived, but it would benefit from a small additional effort to better explain the main claim and its implications.

Does the paper provide a comprehensive overview of the policy and the context of its implementation in a way which is accessible to a general reader?

Yes

Is the discussion on the implications clearly and accurately presented and does it cite the current literature?

Partly

Are the recommendations made clear, balanced, and justified on the basis of the presented arguments?

Yes

Reviewer Expertise:

Public policy analysis and evaluation, infrastructure appraisal, economic evaluation

I confirm that I have read this submission and believe that I have an appropriate level of expertise to confirm that it is of an acceptable scientific standard.

# Peer review: Reviewer response for version 1

**DOI:** 10.5256/f1000research.140729.r164512

**Published:** 2023-03-28

**Authors:** Frédéric Sgard

**Affiliations:** 1 OECD, Paris, France

This policy brief addresses a very important element of research policy, which is the understanding by decision-makers of the role, relevance, and impact of research infrastructures. In this particular case, of research data infrastructure, by using a comparison with a much well known type of infrastructures: roads. This comparison with a much more mundane example (roads) is aptly supported by a strong argument regarding the usefulness of the infrastructure mostly appears when its activity is disrupted, as normal operation is often taken for granted and thus invisible. The comparison between data research infrastructures and roads is clearly and convincingly argumented. This brief is also timely as funders are increasingly stretched to provide support to existing and new research infrastructures and have to make selective choices in their investments.

Does the paper provide a comprehensive overview of the policy and the context of its implementation in a way which is accessible to a general reader?

Yes

Is the discussion on the implications clearly and accurately presented and does it cite the current literature?

Yes

Are the recommendations made clear, balanced, and justified on the basis of the presented arguments?

Yes

Reviewer Expertise:

Science policy

I confirm that I have read this submission and believe that I have an appropriate level of expertise to confirm that it is of an acceptable scientific standard.
